# Photothermal interaction of a 1927 nm Thulium laser with MatriDerm: an in vitro study

**DOI:** 10.1007/s10103-026-04943-z

**Published:** 2026-09-12

**Authors:** Jonathan Herron, JeeHwan Ahn, Mark Brewin

**Affiliations:** 1 Barts Health NHS Trust, London, UK; 2 University of Bolton, Bolton, UK; 3 Imperial College London, London, UK; 4 Salisbury NHS Foundation Trust, Salisbury, UK

**Keywords:** MatriDerm, Thulium laser, Heat shock proteins, Dermal substitutes, Wound healing, Photothermal interaction

## Abstract

Dermal templates support wound healing, yet complex wounds remain challenging. Thermal lasers activate regenerative heat shock pathways through the heat shock response (HSR), but their interaction with dermal substitutes is unclear. MatriDerm^®^, a non-cross-linked collagen-elastin substitute used in single-stage reconstruction, may be enhanced by laser applications to improve regenerative outcomes. This study evaluated the interaction between a 1927 nm Thulium laser and MatriDerm^®^ under different hydration states. MatriDerm^®^ scaffolds (Thickness 1–3 mm) were tested in dry, water-soaked and blood-soaked conditions (*n* = 27). Samples received 1927 nm Thulium laser exposure (20 mJ/ pulse, 10 J/cm^2^, 1 mm spot, five pulses/ site). Macroscopic and microscopic effects, peak temperatures, and charring depth were documented. Blood-soaked scaffolds underwent quantitative polymerase chain reaction (PCR), western blot, enzyme-linked immunosorbent assay (ELISA) and Fourier-transform infrared (FTIR) analysis. Laser effects occurred only in blood-soaked MatriDerm^®^. Dry and water-soaked scaffolds showed no structural change and minimal temperature rise (28–32 °C). Blood-soaked samples reached 65 ± 5 °C, producing superficial carbonisation (250 ± 50 μm), with preserved deeper matrix. Molecular analysis showed HSP70 upregulation, reduced transforming growth factor β1 signalling, increased collagen I expression, and collagen reorganisation. Effects reflected local thermal confinements within blood-rich microdomains rather than bulk absorption at 1927 nm, whereas water alone dissipated heat too rapidly to sustain a photothermal response. 1927 nm Thulium laser produces a distinct photothermal response in the presence of blood, suggesting an altered local optical-thermal environment. These findings support laser-activated dermal scaffolds and provide a mechanistic basis for future in vivo studies exploring Thulium laser-assisted wound healing.

## Introduction

With major reconstructive wounds that are too deep or extensive, primary closure with sutures or local flaps is often not possible. In such cases, dermal substitutes are used to provide a collagen-based scaffold that acts as an extracellular matrix to promote wound healing and tissue regeneration [1]. MatriDerm^®^ (MedSkin Solutions Dr. Suwelack AG, Germany) is a non-cross-linked collagen- elastin matrix and is widely used in single-stage reconstruction, offering pliable coverage. However, certain factors like age, comorbidities, radiation therapy, or technical limitations such as access to the operating room or to the necessary resources can delay the healing process, thus offering a bigger time window in which fibroblasts may undergo the myofibroblast differentiation, promote disorganized collagen deposition and scarring, and even lead to fibrosis and contraction [2, 3]. Further research with MatriDerm^®^ could help us enhance the healing process, optimize the wound closure time and improve functional and cosmetic results even in the more complex wounds.

Lasers are well established in dermatology and scar management. Fractional CO_2_ and Erbium: YAG laser remodel mature hypertrophic scars, while pulsed dye lasers (PDL) reduce erythema and improve pliability [4–6]. More recently, experimental work has shown that thermal lasers can not only act as destructive tools, but as biostimulatory signals by induction of a heat shock response (HSR) that upregulates heat shock proteins (HSPs). This has led to the concept of “laser preconditioning,” where controlled thermal stress primes tissue for improved healing [7].

The non-ablative 1927 nm Thulium laser is conventionally described as a water-absorbing wavelength and is used clinically for pigmentary disorders and skin resurfacing [8]. However, the interaction of the 1927 nm with acellular dermal scaffolds has not been previously characterised.

The 1927 nm Thulium laser was selected for this study because it is the standard non-ablative laser used clinically for skin resurfacing, allowing evaluation of its interaction with MatriDerm^®^ under conditions relevant to a vascularised wound bed. Unlike CO_2_ or Er: YAG lasers, which are ablative at clinical doses and would disrupt the scaffold architecture, the 1927 nm wavelength is non-ablative at moderate parameters. PDL targets oxyhaemoglobin selectively in superficial vasculature through visible wavelength absorption and is not designed for scaffold interaction [9]. Similarly, low-level light therapy (LLLT) and light-emitting diode (LED) devices function at substantially lower power densities (typically mW/cm² rather than kW/cm²) and introduce photobiomodulatory rather than photothermal effects and were outside the scope of this study [10, 11].

MatriDerm^®^ was chosen as the scaffold model because it is one of the most widely used non-cros-linked collagen-elastin dermal substitute in single-stage reconstruction widely available in standardised thicknesses (1–3 mm) [1]. Its acellular collagen-elastin structure allows isolation of photothermal effects on the matrix independent of cellular responses, providing a controlled baseline before proceeding to cellular or in vivo studies.

It is hypothesised that Thulium laser exposure may interact with blood-soaked dermal substitutes in a manner that produces localised thermal shock effects that may trigger regenerative cascades.

This study aimed to evaluate the photothermal interaction of the 1927 nm Thulium laser (Ultra™, Lutronic Corp, South Korea) with MatriDerm^®^ under different hydration states (dry, water-soaked, blood-soaked). By identification of the conditions where there is microscopic and macroscopic change, the aim of this study was to explore the idea of non-ablative laser preconditioning as an adjunct to dermal substitute-based reconstruction.

## Methods

### Sample preparation

MatriDerm^®^ scaffolds (MedSkin Solutions Dr. Suwelack AG, Germany) were prepared in three conditions at thicknesses of 1 mm, 2 mm and 3 mm (*n* = 3 per thickness per group, total *n* = 27). These represent the full range of commercially available thickness and were included to determine whether scaffold thickness influenced the photothermal response, and specifically whether the laser would perforate through the thicker scaffolds.

- Group 1 (Dry): Samples left as packaged.
- Group 2 (Water-Soaked): hydrated in deionised water for 30 min until weight stabilisation and confirmed maximal absorption. Deionised water was used to provide a standardised hydration medium with controlled composition, as the variable ion content of tap water could introduce uncontrolled optical or thermal confounders. Maximal absorption was confirmed by the gravimetric method: scaffolds were weighed at intervals during the soaking period until no further weight increase was observed.
- Group 3 (Blood-soaked): saturated for 30 min with whole blood (haematocrit 42%, defibrinated to prevent clotting), obtained from a single healthy donor (the author), via venepuncture under aseptic technique. Blood was mechanically defibrinated and used fresh. Prior to laser exposure, excess surface fluid – defined as any fluid leaking into the surrounding area when the specimen was positioned for treatment - was blotted before use.

This study used autologous blood from the author in a non-clinical ex vivo laboratory. According to institutional guidelines, formal research ethics approval was not required.

For each hydration group, untreated (non-lased) scaffolds prepared under identical conditions served as controls.

### Laser exposure

Samples were exposed to a 1927 nm Thulium laser (Ultra, Lutronic Corp., South Korea) at 20 mJ per pulse, fluence 10 J/cm^2^, pulse duration 200 µs (0.2 ms), repetition rate 1 Hz, spot size 1 mm, with 5 pulses per site. The peak irradiance was approximately 12.7 kW/cm² and the total emission time per treatment site was 1.0 ms. These parameters represent the standard clinical dosimetry used for non-ablative skin resurfacing with this device. A single standardised parameter set was used to isolate the effect of hydration state. Each sample received a single laser treatment session.

Treatments were performed in a controlled environment at room temperature (22 °C) and humidity 50%. Infrared thermography (FLIR 1320) recorded peak surface temperatures during exposure. Following laser exposure, all samples were immediately photographed (OMAX 5MP Digital USB Microscope Camera) and subjected to infrared thermography. Microscopic features were assessed by haematoxylin and eosin (H&E) staining at 10x and 40x magnification. Charring depth was measured with ImageJ software (National Institutes of Health, USA) as mean ± Standard deviation (SD) in microns from microscopic images.

### Molecular analyses

Blood-soaked scaffolds were incubated 24 h at 37 °C post-laser for molecular assays.

- Quantitative polymerase chain reaction (qPCR): RNA was extracted with TRIzol (Thermo Fisher), reverse-transcribed (SuperScript IV, Invitrogen), and amplified (SYBR Green, Applied Biosystems). Targets included HSPA1A (HSP70), Transforming growth factor-β1 (TGF-β1), COL1A1 (collagen I), normalised to glyceraldehyde 3-phosphate dehydrogenase (GAPDH). Fold changes relative to untreated blood-soaked controls (ΔΔCt method).
- Western blot: Protein lysates (radioimmunoprecipitation assay buffer) were separated on 10% sodium dodecyl sulphate-polyacrylamide gel electrophoresis (SDS-PAGE), transferred to membranes, and probed with antibodies against HSP70, phospho-Smad2/3, collagen I, and β-actin (Abcam) and quantified by densitometry (Image Lab, Bio-Rad).
- Enzyme-linked immunosorbent assay (ELISA): Soluble HSP70 and TGF-β1 levels were quantified (R&D Systems kits, pg/mL).
- Fourier-transform infrared (FTIR) spectroscopy: After 24-hour incubation, blood-soaked scaffolds designed for FTIR analysis were gently rinsed in deionised water and freeze-dried (lyophilised) for 24–48 h until constant weight. FTIR spectra were acquired on the dried scaffolds within 48 h of lyophilisation using a PerkinElmer Spectrum for collagen and spectral shifts (amide I band ~ 1650 cm^−1^).

### Statistical analysis

All data were reported as mean ± SD. One-way analysis of variance (ANOVA) with Tukey’s *post hoc* test was used (GraphPad Prism, GraphPad Software, USA). Significance was set at *p* < 0.05.

## Results

**Fig. 1:**
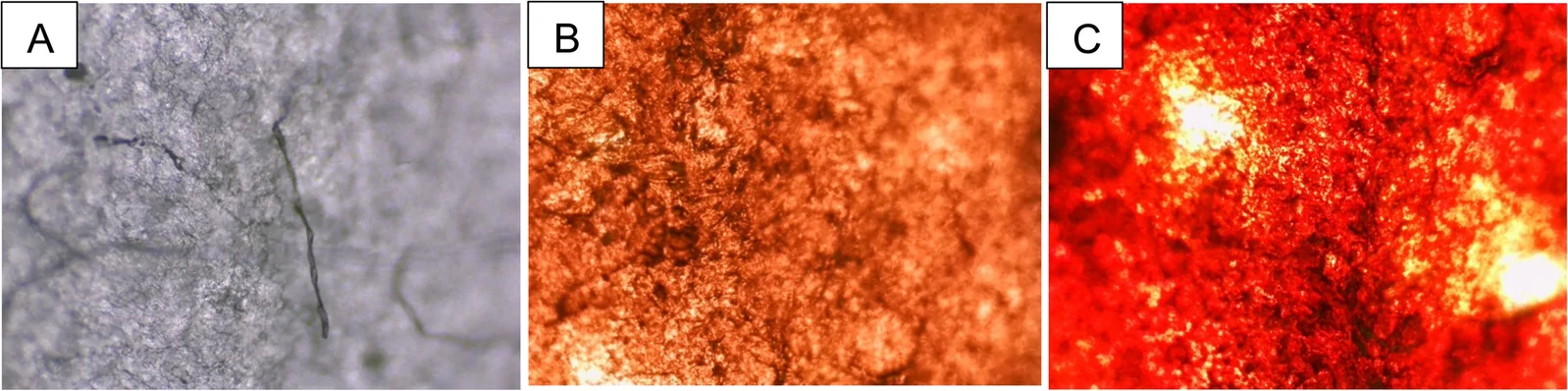
Microscopic images of MatriDerm^®^ samples after Thulium laser exposure. (**A**) Dry scaffold showing no thermal effects and no disruption of collagen architecture. (**B**) Water-soaked scaffold with no disruption of collagen structure. (**C**) Blood-soaked scaffold showing superficial carbonisation

Laser effects differed markedly between hydration states, with no differences across scaffold thickness (*p > 0.05*). Representative microscopic findings are shown in Fig. 1. Thermal and histological findings are summarised in Table 1.

**Table 1:** Macroscopic and microscopic findings following thulium laser exposure

Group	Peak temperature (°C)	Charring depth (µm)	Histology (H&E, 40x)
Dry	28 ± 2	0	Intact, uniform fibres
Water-soaked	32 ± 3	0	Intact, uniform fibres
Blood-soaked	65 ± 5	250 ±50	Superficial carbonisation, deeper structures intact on H&E but not formally characterised

### Molecular effects in blood-soaked scaffolds

Molecular changes were only observed in blood-soaked scaffolds exposed to laser; dry and water-soaked samples showed no measurable changes (all < 1.1-fold, *p* > 0.05) (Table 2).

**Table 2:** Molecular findings in blood-soaked scaffolds after laser exposure

Assay	Marker	Fold change/Level (Laser vs. Control)	*P*-value	Interpretation
qPCR	HSP70	2.5 ± 0.4-fold	< 0.01	HSR activation
qPCR	TGF-β1	0.7 ± 0.1-fold	< 0.05	Reduced pro-fibrotic signalling
qPCR	COL1A1	1.6 ± 0.2-fold	< 0.05	Enhanced collagen expression
Western	HSP70	2.1 ± 0.3-fold	< 0.01	Confirms HSP70 protein increase
Western	p-Smad2/3	0.6 ± 0.1-fold	< 0.05	Attenuated TGF-β pathway
Western	Collagen I	1.4 ± 0.2-fold	< 0.05	Matrix protein upregulation
ELISA	HSP70	120 ± 15 (vs. 45 ± 10)	< 0.01	Soluble chaperone increased
ELISA	TGF-β1	85 ± 12 (vs. 140 ± 18)	< 0.05	Reduced cytokine release
FTIR	Amide I shift	−7 cm^− 1^	NA	Collagen structural reorganisation

## Discussion

This study demonstrates that the 1927 nm Thulium laser interacts with MatriDerm^®^ in a hydration-dependent manner, where observable changes were seen exclusively when the matrix was saturated with blood. There were no visible alterations on the dry or water-soaked samples following laser exposure. These findings indicate that the presence of blood fundamentally changes the optical-thermal environment of the scaffold, though the precise mechanism responsible for this differential response remains unresolved [12].

### Laser tissue interaction and thermal shock

The mid-infrared 1927 nm corresponds to a strong peak of water absorption, where is it absorbed more strongly than at the near-infrared wavelength (e.g. 1550 nm erbium: glass), yet less than the absorption seen with CO_2_ (10,600 nm) or Er: YAG (2940 nm) lasers [13, 14]. This balance means that Thulium laser is able to achieve an intermediate penetration depth of approximately 200–300 μm, with energy confined within the superficial dermis [13, 15]. For this reason, the 1927 nm laser is widely used in dermatology for pigmentation disorders, melasma and skin resurfacing, where it can remove melanin-containing keratinocytes and stimulate dermal remodelling whilst preserving deeper structures [14, 16, 17].

In clinical skin, published optical coherence tomography studies at 1927 nm using 20 mJ pulse energy report microthermal treatment zones extending to approximately 200–400 μm [14]. In our acellular MatriDerm^®^ model, the observed carbonised depth (250 ± 50 μm) falls within this range, though the penetration depth in a porous scaffold lacking intact epidermis and dermis may differ from that in intact skin due to differences in water content, scattering and tissue architecture.

At 1927 nm, water is the dominant chromophore, with absorption greater than in the near infrared, but lower than that at the fully ablative CO_2_ or Er: YAG wavelength [18]. This results in energy confinement to the superficial 200–300 μm, corresponding in clinical skin to the epidermis and upper papillary dermis. Melanin is localised to the epidermis and plays no role at this wavelength. Haemoglobin, the principal chromophore of dermal vasculature, absorbs strongly between 400 and 600 nm but contributes comparatively little above 1000 nm where water absorption predominates [18, 19].

However, when whole blood is present in concentrated form within a porous scaffold rather than confined within vessels, its composite properties such as protein content, viscosity, scattering may alter the local photothermal response beyond what chromophore absorption alone would predict.

 In vivo, tightly packed epidermal cells allow confined energy deposition in water and can lead to controlled ablation, collagen stimulation and growth factor upregulation associated with skin renewal [17]. However, in our ex vivo MatriDerm^®^ model, water alone did not produce visible changes. This may be because the unconfined collagen elastin scaffold allowed rapid dissipation of heat. By comparison, blood saturation introduced additional chromophores and altered the thermal transport properties of the scaffold, enabling focal deposition of energy and rapid temperature elevation to produce superficial carbonisation while preserving deeper structures. This observation is consistent with a study where Thulium laser at adjacent wavelengths (1940 nm) achieved precise vascular coagulation in neurovascular models with no ablation, supporting the relevance of haemoglobin-related absorption in this spectral range, though direct comparison with our acellular model should be made cautiously [20].

It is important to note that the observed difference between water-soaked and blood-soaked conditions cannot be attributed solely to haemoglobin absorption. At 1927 nm, water absorption dominates the tissue absorption spectrum and haemoglobin absorption is comparatively low [18]. Other factors may contribute to the differential response, including: the higher viscosity of blood compared with water, which may reduce fluid redistribution and heat dissipation within the porous matrix; the protein content of blood (haemoglobin, albumin and other plasma proteins), which introduces additional absorbing species and alters scattering properties; differences in fluid distribution within the scaffold pores; and local confinement of heated microdomains due to the complex behaviour of blood within a porous collagen-elastin network. As such, the observed effect may arise from a combination of optical and thermal factors rather than a single dominant absorber.

### Thermal relaxation time

Although 1927 nm is commonly described as “water absorbing,” absorption alone does not guarantee visible change and rather, the key determinant is whether absorbed energy remains thermally confined long enough to produce structural effects. The concept of thermal relaxation time (TRT), the time required for heated tissue to cool to 50% of its peak temperature after laser exposure, provides a framework for understanding this, though we note that the thermal behaviour of a porous collagen-elastin scaffold is complex and likely depends not only on wavelength and absorption, but also on pore structure, fluid occupancy, heat diffusion paths, and pulse timing [21, 22].

Water has a short TRT and rapidly conducts heat away and so in an acellular hydrated matrix, deposited heat may have rapidly dissipated throughout the scaffold and prevented the temperature from remaining above the critical threshold (42–45 °C) required to activate the heat shock response and denature collagen. However, in the blood-soaked group, the combination with the chromophore content, higher viscosity and altered thermal transport, may have created areas of thermal confinement where the rate of energy deposition was greater than that of thermal diffusion. This persisted long enough to induce microthermal elevations (65 °C) sufficient to cause carbonisation and trigger a heat shock response. This mechanism is consistent with prior optical-thermal models showing that chromophore-rich structures such as blood vessels retain heat several times longer than surrounding stroma, allowing selective photocoagulation without widespread tissue injury [23].

Additionally, this principle is comparable to the “activated fibre tip” used in diode laser systems, where localised chromophore absorption leads to efficient photothermal conversion, creating confined microdomains of elevated temperature to a small volume [24]. Similarly, in the blood soaked MatriDerm^®^ blood may have provided localised absorption centres that concentrated energy whereas the water dissipated heat too rapidly to sustain such confinement.

The thermal confinement concept presented here is plausible but remains to be confirmed through formal optical-thermal modelling. Understanding the differences in parameters of thermal diffusion and relaxation kinetics rather than energy delivery alone is essential for optimising pulse duration and fluence in future translational studies.

### Heat shock response

An important implication of our findings is the potential induction of a heat shock response (HSR) following Thulium laser exposure in vascularised tissue. Regardless of wavelength, thermal lasers generate physiological temperature gradients in the surrounding dermis, with 42 °C or above enough to trigger the HSR [25]. This can upregulate heat shock proteins (HSPs), particularly HSP70, which can act as molecular chaperones to stabilise unfolded proteins, promote refolding, and protect cells from apoptosis. In essence, the laser’s controlled thermal stress did not act as an ablative injury, but as a biological signal, alerting cells to activate their intrinsic repair machinery.

HSP 70 has been shown to interact with TGF-β, a key regulator of inflammation and fibrogenesis. By stabilising major signalling pathways, the HSR may dampen excessive pro-inflammatory cascades, attenuating mediators such as TNF-a, IL-1β, and inducible nitric oxide synthase, while promoting an environment conducive to regeneration [26]. Kovalchin et al. indicated that exogenous HSP70 accelerated wound closure in murine models by upregulating macrophage phagocytosis and stimulating cytokine release, including platelet derived growth factors and vascular endothelial growth factors [27]. Immunohistochemical studies of acute wounds have confirmed upregulation of HSP27, HSP60, HSP70 and HSP90 while chronic or impaired wounds exhibit reduced HSP expression [28, 29].

The fact that visible changes in our study occurred only in blood-soaked MatriDerm^®^ provides a plausible physical foundation for hypothesising that Thulium laser irradiation of vascularised dermal substitutes may induce HSP activity in vivo, triggering regenerative cascades that improve healing outcomes.

### Fibroblast modulation and scar prevention

Fibroblasts play a central role in dermal repair, synthesising collagen and extracellular matrix. Under profibrotic conditions, these cells differentiate into myofibroblasts, whose contractile phenotype is associated with hypertrophic scarring, keloids and contracture [30, 31]. MatriDerm^®^ is composed of non-cross-linked collagen, unlike other dermal substitutes that undergo cross-linking to increase structural stability. Its more compliant architecture provides a scaffold that promotes regenerative fibroblast behaviour and reduces fibroblast to myofibroblast transition, thereby reducing the risk of contracture [2, 3]. Our molecular data, showing HSP70 upregulation coupled with reduced TGF- β1/Smad signalling and increased collagen I expression, are consistent with a shift away from profibrotic signalling, though this was observed in an acellular system and the presence of responsive fibroblasts would be required to confirm functional relevance.

The concept that dermal substitutes act synergistically with adjunctive stimuli is supported by existing literature. Hamuy et al. reported that one stage skin grafting with artificial dermis and basic fibroblast growth factor improved scar elasticity and maturation compared to conventional two stage techniques [32]. Xie et al. used an engineered dermal substitute with mesenchymal stem cells (MSC) for wound closure in mice, with higher MSC survival, increased macrophage infiltration and enhanced wound repair [33]. Darwish et al. evaluated Pelnac combined with platelet-rich plasma reporting faster neovascularisation, improved graft safety and shorter hospital stays without compromising scar quality or complications [34]. Although no such study has used laser adjunct to dermal substitute, these reports collectively reinforce the principle that engineered dermal substitutes are receptive platforms for augmentation.

### Lasers in scar modulation

Lasers are well established in scar management, particularly fractional CO_2_ and erbium: YAG devices, which remodel established hypertrophic scars through controlled microthermal injury and collagen remodelling. Pulsed dye lasers have also shown efficacy in reducing erythema, improving pliability, and softening scar tissue, as well as modulating fibroblast behaviour to reduce tissue contraction without causing damage [35, 36]. Brewin et al. suggested that lasers may also have a preventative role [37]. Ibrahim et al. saw improved long-term scar quality in early application of fractional CO_2_ compared with delayed treatment, typically 4–6 weeks post-surgery when scar formation is premature [38].

Our finding suggests that laser interaction with dermal scaffolds during the initial reconstructive phase, rather than after scar formation, may represent a complementary approach, though this remains to be tested in vivo.

### Limitations

This study has several limitations. Firstly, it utilised an acellular, ex vivo scaffold model, which does not capture cellular response or the complexity of in vivo wound environments. Blood samples were obtained using a single donor and a single set of laser parameters was tested. Additionally, the microscope calibration was not recorded at the time of image acquisition. The estimated field view is approximately 200 μm, but validated scale bars could not be added. Similarly, the depth of preserved structures below the carbonisation zone was observed but not formally characterised.

### Future directions

Future studies should explore the role of pulse structure, repetition rate, and scaffold hydration in modulating thermal confinement, alongside optical-thermal modelling and validation in cellular and in vivo wound models.

## Conclusion

This in-vitro study demonstrates that blood-soaked MatriDerm^®^ exhibits a distinct photothermal response to 1927 nm Thulium laser exposure, characterised by localised thermal elevation and associated structural and molecular changes, whereas dry and water-soaked conditions do not. The differential response reflects changes in the scaffold’s optical-thermal environment introduced by blood, although the precise mechanisms remain unresolved, and the relative contributions of chromophore absorption, thermal transport, and scaffold-fluid interaction require further investigations. These results provide a basis for further investigations of laser-dermal substitute interactions under physiologically relevant conditions.

## Data Availability

All data generated or analysed during this study are included within the article.
